# Optimal Caliper Width for Propensity Score Matching of Three Treatment Groups: A Monte Carlo Study

**DOI:** 10.1371/journal.pone.0081045

**Published:** 2013-12-11

**Authors:** Yongji Wang, Hongwei Cai, Chanjuan Li, Zhiwei Jiang, Ling Wang, Jiugang Song, Jielai Xia

**Affiliations:** 1 Medical Department, The 309th Hospital of Chinese People's Liberation Army, Beijing, China; 2 Department of Health Statistics, The Fourth Military Medical University, Xi'an, Shaanxi, China; 3 Information Center, School of Stomatology, The Fourth Military Medical University, Xi'an, Shaanxi, China; 4 Department of Gastroenterology, The 309th Hospital of Chinese People's Liberation Army, Beijing, China; Cardiff University, United Kingdom

## Abstract

Propensity score matching is a method to reduce bias in non-randomized and observational studies. Propensity score matching is mainly applied to two treatment groups rather than multiple treatment groups, because some key issues affecting its application to multiple treatment groups remain unsolved, such as the matching distance, the assessment of balance in baseline variables, and the choice of optimal caliper width. The primary objective of this study was to compare propensity score matching methods using different calipers and to choose the optimal caliper width for use with three treatment groups. The authors used caliper widths from 0.1 to 0.8 of the pooled standard deviation of the logit of the propensity score, in increments of 0.1. The balance in baseline variables was assessed by standardized difference. The matching ratio, relative bias, and mean squared error (MSE) of the estimate between groups in different propensity score-matched samples were also reported. The results of Monte Carlo simulations indicate that matching using a caliper width of 0.2 of the pooled standard deviation of the logit of the propensity score affords superior performance in the estimation of treatment effects. This study provides practical solutions for the application of propensity score matching of three treatment groups.

## Introduction

PSM (propensity score matching) is widely used to reduce bias in non-randomized and observational studies [1], [2], [3]. The propensity score(PS), introduced by Rosenbaum and Rubin in 1983 [4], is defined as a subject's probability of receiving a specific treatment conditional on a group of observed covariates. As the representation of many covariates, it is estimated at baseline to control selection bias. There are four main propensity score methods—propensity score matching, stratification on propensity score, covariate adjustment using propensity score, and propensity score weighting [5]—among which PSM is used most commonly [6].

Propensity score methods have been widely applied to two treatment groups, but few studies has reported its use for multiple treatment groups. Imbens extended Rosenbaum and Rubin's work to multiple treatment groups. The multiple propensity score, defined as the probability of receiving a particular treatment conditional on the observed covariates, can be estimated by a multinomial logistic regression, given that there is no inherent order among the different treatments [7]. Wang et al. proposed the application of stratification on the multiple propensity score to dose-response relationships in drug safety studies [8]. With a practical step-by-step approach using data from a mental health study, Spreeuwenberg et al. introduced covariate adjustment using multiple propensity score [9]. However, none of the existing studies, as far as we know, had dealt with PSM for multiple exposure groups. Many key issues have not been resolved, such as the assessment of balance in baseline variables and sensitivity analysis, which limit the application of multiple PSM. In addition, how to select the optimal caliper is another key issue in multi-treatment PSM. Austin summarized eight caliper widths commonly employed in two treatment group scenario [10]. Two or three treatment groups are common in clinical practice. The primary objective of this study was to choose the optimal caliper for three treatment groups by comparing the PSM methods of different calipers based on Monte Carlo simulations.

## Methods

### 2.1. Theory

Two different approaches of matching are available in PSM: global optimal algorithms and local optimal algorithms (also referred to as greedy algorithms) [11]. Global optimal algorithms use network flow theory, which can minimize the total distance within matched subjects [12]. Global methods may be difficult to implement when there are large numbers of potential controls from which to choose, since they require the creation of a large distance matrix. Local optimal algorithms begin with the random selection of the first subject in the treatment group, and then find its closest control match based on the absolute value of the difference between propensity scores (or the logit of the scores). We chose to use local optimal algorithms for PSM among three treatment groups, as is done commonly for two treatment groups. The simulations were all based on 1∶1∶1 matching within a pre-specified caliper width, without replacement. In this schema, the first subject in group-1 is matched with subjects from group-2 and group-3 using the smallest distance. The caliper width defines the range within which the propensity scores (or logit of the propensity scores) must fall to be considered a valid match [13]. Wider caliper widths can result in the inclusion of more subjects, a greater sample size, and more precision, but can also decrease balance between groups and introduce more bias in estimating treatment effects. The opposite is true for narrower caliper widths. Thus, only those subjects with distances fallen within the caliper width are being included. Those subjects not matched are supposed as outliers and being excluded. ATE (Average Treatment Effect) and ATT (Average Treatment Effect for the treated) are commonly estimated based on potential outcomes, or so called counterfactual outcomes [14]. ATE is a weighted average of ATT and ATU (Average Treatment Effect for the untreated). In PSM using caliper with two treatments or more, only sufficiently overlapped regions among groups were considered, that is to say, to match only those units in one group with covariate values that are sufficiently close to the values observed in other groups. When this is done, the quantity estimated is no longer the ATE or ATT, because the average is only taken over the region of common support. However, we can call it average treatment effects for the matched samples.

The sum of the probabilities of a subject receiving treatment *g* is defined as:
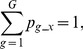
(1)where *p_g_*__x_(*g* = 1,2…*G*) denotes the subject *x*'s probability of receiving *g*-th group treatment conditional on the observed covariates, and *G* denotes the number of the group. For two treatment groups, the variance of the propensity score of the treatment group is equal to the variance of the propensity score of the control group, and therefore considering the probability of the subject receiving one of the two treatments is sufficient.

When considering more than two treatment groups, however, the variances of the propensity score of *g* groups are not equal. Therefore, *g* propensity scores should be taken into account for the purpose of matching.

For two treatment groups, the matching distance D can be defined either by D_1_ or D_2_:
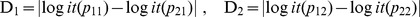
(2)
*p*_11_ is the probability of receiving treatment 1 conditional on the observed covariates for the patient in treatment 1, *p*_21_ is the probability of receiving treatment 1 conditional on the observed covariates for the patient in treatment 2, and etc.
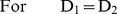


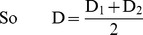
(3)


For three treatment groups, each patient has three Propensity Scores. Notation of Propensity Scores in 3 treatment groups is presented in Table 1.

**Table 1:** Notation of Propensity Scores in 3 treatment groups.

Treatment(Actual)	Treatment_1_	Treatment_2_	Treatment_3_
Treatment_1_	*p*_11_	*p*_21_	*p*_31_
Treatment_2_	*p*_12_	*p*_22_	*p*_32_
Treatment_3_	*p*_13_	*p*_23_	*p*_33_

For three treatment groups, formula (3) can be extended as:
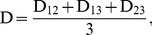
(4)

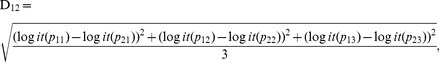
(5)

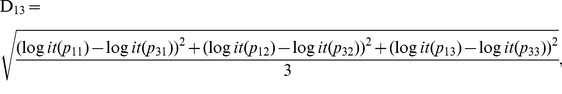
(6)

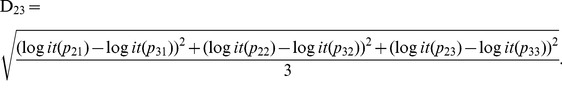
(7)


For multi-treatment groups, the matching distance D can be extended as:
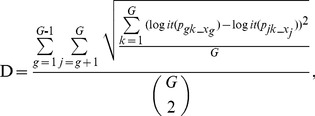
(8)where ***p_gk_x_***(*g* = 1,2…*G*-1; *k* = 1,2…*G*) denotes the *g*-th group subject ***x_g_*** **'s** probability of receiving the *k-th* group treatment conditional on the observed covariates, and ***p_jk_x_***(*j* = *g*+1,*g*+2…*G*; *k* = 1,2…*G*) denotes the *j*-th group subject ***x_j_*** **'s** probability of receiving the *k-th* group treatment conditional on the observed covariates. Propensity score matching methods have not been used for three treatment groups in prior empirical research, and no commonly used caliper widths can be referenced. We allowed caliper widths to range from 0.1 to 0.8 of the pooled standard deviation of the logit of the propensity score in increments of 0.1 using Monte Carlo simulations. For multi-treatment groups, the pooled standard deviation is defined as:
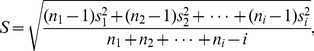
(9)where *S_i_* denotes the standard deviation of the logit of the propensity score, and *i* is the number of the sample combined.

Another key issue in this study is the evaluation of the balance of baseline covariates before and after PSM. The balance of baseline variables should be assessed by the standardized difference, which was first introduced by Flury and Reidwyl in 1986 [15]. Its use has recently become common in propensity score studies [16], [17]. Standardized difference can be used for both continuous and dichotomous variables. For continuous variables, the standardized difference is defined as:
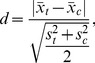
(10)where 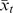 and 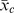 denote the mean of the variable in treatment and control subjects, respectively, and 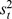 and 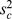 denote the variances of the variable in the treatment and control groups, respectively. For dichotomous variables, the standardized difference is defined as:
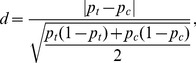
(11)where 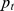 and 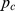 denote the proportion of treatment and control groups, respectively. It has been suggested that a standardized difference of less than 0.1 indicates negligible imbalance in a given covariate between groups [18]. Standardized difference satisfies the two properties of balance assessment methods proposed by Imai et al. [19]. First, it should be a characteristic of the sample rather than a characteristic of the population. Second, this statistic should be unaffected by sample size. Hypothesis testing does not satisfy these two properties. A statistical test of hypothesis is influenced by the sample size, and as a result, better balance may be achieved in the matched samples than in the initial overall sample due simply to a smaller sample size. As of now, standardized difference has only been applied in the analysis of two treatment groups. For multiple treatment groups in this study, pairwise comparison is conducted to calculate standardized difference, among which the greatest standardized difference is chosen as an indicator to access the overall balance in our study.

### 2.2. Monte Carlo Simulations

We used Monte Carlo simulations to examine the performance of the eight different calipers.

#### 2.2.1

We generated eight imbalanced variables among three treatment groups—4 continuous variables (*C*_1_, *C*_2_, *C*_3_, and *C*_4_) and 4 dichotomous variables (*D*_1_, *D*_2_, *D*_3_, and *D*_4_ [7])—and assumed that one continuous covariate and one dichotomous covariate had maximal standardized differences of 0.2 among the three groups. Similarly, we assumed that the remaining 3 pairs of continuous and dichotomous variables had maximal standardized differences in the full sample of 0.3, 0.4, and 0.5. The 8 variables are independent.

For each of the 1000 subjects, *T_j_* (*j* = 1, 2, 3) denotes a multi-nominal grouping variable. *T_j_* was transformed to a set of dummy variables where *T*_3_ was considered as the base category (control group) as follows:
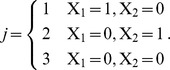
Continuous covariates were generated randomly from the following distribution:
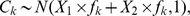
where *C_k_* denotes the *k*-th continuous covariate. Thus, the distribution of the continuous covariates would be *N*(*f_k_*,1) for group-1 subjects and group-2 subjects, and *N*(0,1) for group-3 subjects. The values of *f_k_* were set as 0.25, 0.35, 0.45, and 0.55, so that the maximal value of the four pairs of continuous covariates had standardized differences of 0.2, 0.3, 0.4, and 0.5 among the three groups (this was determined in an initial set of Monte Carlo simulations).The prevalence of the 4 dichotomous variables among group-3 subjects was taken to be 0.1, 0.2, 0.3, and 0.4. The prevalence of the 4 dichotomous variables among the exposed subjects was selected so that the maximal standardized differences of the 4 dichotomous variables among the three groups were 0.2, 0.3, 0.4, and 0.5. This was achieved by setting the prevalence of the 4 dichotomous variables among group-1 subjects to be 0.147, 0.312, 0.475, and 0.612, respectively. Prevalence of the 4 dichotomous variables among group-2 subjects were set to the same values (this was determined from an initial set of Monte Carlo simulations).

#### 2.2.2

Once we had randomly generated the treatment status, 4 continuous variables and 4 dichotomous variables for each of the 1000 subjects, we randomly generated a continuous outcome for each subject using the following linear model:
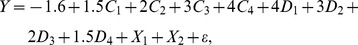
(12)where 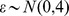, where *X*_1_ and *X*_2_ denote the treatment status. The coefficients of the 4 continuous variables and 4 dichotomous variables denote different correlations with outcome from weak to strong. The results of normality test for outcome Y are presented in Table 2. The outcome Y obeys normal distribution in the scenarios we considered.

**Table 2:** Results of Normality Test for Outcome Y.

caliper	*P*		
	pre_matching ratios of subjects: 1∶2∶7	pre_matching ratios of subjects: 1∶2∶3	pre_matching ratios of subjects: 2∶3∶5
unmatched	0.148	0.052	0.052
10%	0.091	0.097	0.088
20%	0.105	0.119	0.109
30%	0.112	0.12	0.103
40%	0.103	0.115	0.103
50%	0.104	0.106	0.102
60%	0.106	0.102	0.089
70%	0.106	0.103	0.089
80%	0.108	0.099	0.085

#### 2.2.3

We randomly generated 1000 datasets with the required treatment effect using the above data-generating process (each randomly generated dataset consisted of 1000 subjects with required conditions). Within each randomly generated dataset, we estimated the propensity score by regressing treatment status on the 8 variables using a multinomial logistic regression model. The matched samples were obtained by matching subjects on the logit of the propensity score using nearest neighbor matching, with calipers ranging from 0.1 to 0.8 of the pooled standard deviations of the logit of the propensity score in increments of 0.1. The matching distance was described in Section 2.

#### 2.2.4

The standardized difference(SD) between groups, matching ratio, relative bias(RB), and Mean Square Error(MSE) were applied to assess simulation results. Standardized difference is used for comparing the balance of matched samples. The matching ratio, defined as the number of matching subjects vs. total subjects in the treatment with the least number of subjects, provides an estimate of precision. The relative bias provides a measure of the magnitude of the bias. The smaller the relative bias, the smaller the bias of the propensity score model. The relative bias is defined as:
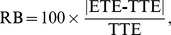
where ETE and TTE denote the estimated treatment effect and true treatment effect, respectively. TTE is taken from the simulation model. ETE is average treatment effects for the matched samples. Group-3 was considered a referenced category(control group), and the relative bias values between group-3 and other groups were reported. Under the data-generating process, the true treatment effect is equal to 1when evaluating group-1 vs. group-3, and also when evaluating group-2 vs. group-3.

The MSE is used to evaluate the precision of the propensity score model, which is defined as:
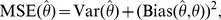
where 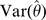 denotes the variance of the estimator, and 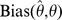 denotes the bias of the estimator. Group-3 was considered the referenced category, and the MSEs between group-3 and other groups were reported. MSE is a quantitative measure of the trade-off between variance and bias. Our focus on MSE allows an investigator to select a caliper width that optimizes this implicit trade-off.

#### 2.2.5

We considered three scenarios, where the pre_matching ratios of subjects (treatment 1: treatment 2: treatment 3) were assumed as 1∶2∶7, 1∶2∶3 and 2∶3∶5, respectively. The “pre_matching ratio” means the predefined ratio of subjects' numbers among three treatment groups before PSM.

All simulations were performed in SAS, version 9.1.

## Results

The mean standardized difference for each of the 8 variables, matching ratio, relative bias, and MSE are presented in Tables 3, 4, 5. RB_13_ denotes the relative bias between group-1 and group-3, and RB_23_ denotes the relative bias between group-2 and group-3. Similarly, MSE_13_ denotes the MSE between group-1 and group-3, and MSE_23_ denotes the MSE between group-2 and group-3.

**Table 3:** Results of Monte Carlo Simulations (pre_matching ratios of subjects: 1∶2∶7).

caliper	Standardized difference for covariates								Matching ratio	RB_13_	RB_23_	MSE_13_	MSE_23_
	C_1_	C_2_	C_3_	C_4_	D_1_	D_2_	D_3_	D_4_					
10%	0.127	0.121	0.120	0.116	**0.129**	0.120	0.110	0.112	82.6%	1.6%	−1.6%	0.306	0.313
20%	0.110	0.106	0.107	0.100	0.111	0.107	0.098	0.101	95.0%	4.8%	−0.4%	0.266	0.273
30%	0.108	0.105	0.105	0.098	0.110	0.105	0.095	0.099	97.8%	8.5%	1.3%	0.265	0.263
40%	0.108	0.105	0.104	0.098	0.110	0.105	**0.094**	0.097	98.9%	11.8%	2.6%	0.269	0.260
50%	0.108	0.105	0.104	0.098	0.110	0.105	**0.094**	0.097	99.4%	14.1%	3.6%	0.276	0.262
60%	0.108	0.104	0.104	0.098	0.110	0.106	**0.094**	0.097	99.7%	15.7%	4.2%	0.283	0.263
70%	0.107	0.104	0.104	0.098	0.111	0.106	**0.094**	0.097	99.9%	16.7%	4.6%	0.289	0.262
80%	0.107	0.104	0.104	0.098	0.111	0.106	**0.094**	0.097	99.9%	17.3%	4.8%	0.293	0.262

**Table 4:** Results of Monte Carlo Simulations (pre_matching ratios of subjects: 1∶2∶3).

caliper	Standardized difference for covariates								Matching ratio	RB_13_	RB_23_	MSE_13_	MSE_23_
	C_1_	C_2_	C_3_	C_4_	D_1_	D_2_	D_3_	D_4_					
10%	0.092	0.092	0.090	0.085	**0.096**	0.089	0.086	0.082	81.5%	4.3%	3.4%	0.179	0.201
20%	0.084	0.082	0.081	0.078	0.087	0.081	0.078	0.073	90.3%	9.2%	5.0%	**0.164**	**0.175**
30%	0.083	0.082	0.079	0.077	0.086	0.081	0.077	**0.072**	92.9%	16.9%	8.1%	0.183	0.176
40%	0.082	0.082	0.080	0.078	0.086	0.082	0.078	**0.072**	94.7%	26.2%	12.5%	0.225	0.182
50%	0.082	0.083	0.081	0.080	0.088	0.085	0.079	0.073	96.2%	35.6%	16.3%	0.288	0.193
60%	0.081	0.083	0.082	0.083	0.089	0.085	0.080	0.074	97.4%	43.8%	19.9%	0.365	0.206
70%	0.081	0.083	0.083	0.085	0.089	0.088	0.082	0.075	98.3%	50.9%	22.7%	0.444	0.220
80%	0.082	0.084	0.085	0.087	0.089	0.089	0.085	0.076	98.9%	56.7%	24.9%	0.516	0.230

**Table 5:** Results of Monte Carlo Simulations (pre_matching ratios of subjects: 2∶3∶5).

caliper	Standardized difference for covariates								Matching ratio	RB_13_	RB_23_	MSE_13_	MSE_23_
	C_1_	C_2_	C_3_	C_4_	D_1_	D_2_	D_3_	D_4_					
10%	0.083	0.081	0.077	0.077	**0.088**	0.080	0.073	0.070	77.3%	3.3%	2.0%	0.152	0.160
20%	0.076	0.075	0.070	0.069	0.079	0.071	0.066	**0.065**	87.0%	8.4%	3.7%	**0.140**	**0.144**
30%	0.076	0.075	0.070	0.069	0.079	0.072	0.068	**0.065**	90.0%	17.4%	7.4%	0.161	**0.139**
40%	0.076	0.074	0.070	0.070	0.079	0.073	0.069	0.066	92.2%	28.5%	12.1%	0.213	0.145
50%	0.076	0.075	0.073	0.074	0.080	0.076	0.072	0.068	94.1%	40.4%	17.5%	0.303	0.160
60%	0.076	0.076	0.075	0.079	0.080	0.079	0.076	0.069	95.6%	52.1%	22.7%	0.424	0.183
70%	0.076	0.077	0.078	0.083	0.082	0.082	0.079	0.072	96.9%	62.2%	27.2%	0.552	0.209
80%	0.076	0.078	0.081	**0.088**	0.084	0.085	0.083	0.075	97.9%	71.4%	31.4%	0.690	0.237

When the pre_matching ratio of subjects was 1∶2∶3, the standardized differences for the 8 variables ranged from 0.072 to 0.096.When the pre_matching ratio of subjects was 2∶3∶5, the corresponding range was from 0.065 to 0.088 for the 8 different caliper widths. In both scenarios a standardized difference of each covariate of less than 0.1 indicates negligible imbalance between treatment groups. When the pre_matching ratio of subjects was 1∶2∶7, the corresponding range was from 0.094 to 0.129, and only 31.3% of the standardized differences were less than 0.1.

Irrespective of the pre_matching ratio of subjects, using a caliper width of 0.8 of the pooled standard deviation of the logit of the propensity score resulted in the greatest matching ratio. When the pre_matching ratios of subjects were 1∶2∶7, 1∶2∶3 and 2∶3∶5, the matching ratios were 99.9%, 98.9%, and 97.9%, respectively. Using caliper width of 0.1 of the pooled standard deviation of the logit of the propensity score resulted in the lowest matching ratio. Thus, when the pre_matching ratios of subjects were 1∶2∶7, 1∶2∶3 and 2∶3∶5, the matching ratios were 82.6%, 81.5%, and 77.3%, respectively.

As seen from matching ratios, using a caliper width of 0.8 of the pooled standard deviation of the logit of the propensity score resulted in the greatest bias, irrespective of the pre_matching ratio of subjects. When the pre_matching ratios of subjects were 1∶2∶7, 1∶2∶3 and 2∶3∶5, the relative biases were 17.3%, 56.7%, and 71.4%, respectively, between group-1 and group-3. Those between group-2 and group-3 were 4.8%, 24.9%, and 31.4%, respectively. Using a caliper width of 0.1 of the pooled standard deviation of the logit of the propensity score resulted in the lowest bias. For the pre_matching ratios of 1∶2∶7, 1∶2∶3 and 2∶3∶5, the relative biases were 1.6%, 4.3% and 3.3%, respectively, between group-1 and group-3, and the relative biases were-1.6%, 3.4% and 2.0%, respectively, between group-2 and group-3.

When the pre_matching ratio of subjects was1∶2∶3 or 2∶3∶5, using a caliper width of 0.2 of the pooled standard deviation of the logit of the propensity score resulted in the lowest MSE between group-1 and group-3. When the pre_matching ratio of subjects was 1∶2∶3, using a caliper width of 0.2 of the pooled standard deviation of the logit of the propensity score resulted in the lowest MSE between group-2 and group-3. When the pre_matching ratio of subjects was 2∶3∶5, using a caliper width of 0.2 of the pooled standard deviation of the logit of the propensity score resulted in a MSE that was negligibly higher than the lowest MSE between group-2 and group-3. When the pre_matching ratio of subjects was 1∶2∶7, the MSE was only slightly lower than that of the highest MSE (MSE ranging from 0.260 to 0.313). When the pre_matching ratio of subjects was1∶2∶3 or 2∶3∶5, using a caliper width of 0.8 of the pooled standard deviation of the logit of the propensity score resulted in the highest MSE.

## Empirical Study

We applied the matching schemes to a perspective observational clinical study designed to evaluate the mortality and morbidity of patients with heart failure already receiving optimal medical therapy in China. There were 601 patients recruited in this study, who were divided into 3 treatment groups by heart failure durations, including less than 1 year (n = 215), 1–5 years (n = 242) and more than 5 years (n = 144).

In order to reduce the bias from confounding variables, PSM was used to adjust the baseline differences. Standardized differences for covariates before and after matching is compared (We can't calculate the RB and MSE in this example because the average treatment effect is unknown). The propensity score was estimated by using a logistic regression model. Heart failure durations were used as the dependent variable, and the other 9 confounders that were identified in Table 6 as independent variables, including sex, rhythm, LVEDD (left ventricular end-diastolic dimension), age, NYHA (New York Heart Association) class, QRS duration, LVEF (Left Ventricular Ejection Fraction), Heart rate and Medicine therapy. We used 0.2 of the pooled standard deviation of the logit of the propensity score as caliper width for PSM. The matching ratio was 73.6%. The balances of baseline variables were assessed by standardized difference. The standardized differences for the 9 confounders were all bigger than 0.1 before PSM. The standardized differences of all confounders were smaller than 0.1 except Medicine therapy, which indicates negligible imbalance among treatment groups after PSM.

**Table 6:** Results of Empirical Study.

caliper	Standardized difference for covariates									Matching ratio
	sex	rhythm	LVEDD	age	NYHA	QRS	LVEF	Heart rate	Medicine therapy	
unmatched	0.214	0.387	4.288	0.276	0.317	0.407	0.203	0.156	0.292	—
20%	0.100	0.053	0.027	0.088	0.050	0.098	0.043	0.094	0.142	73.6%

## Discussion

The primary objective of the current study was to compare the PSM methods of different calipers and to choose the optimal caliper for three treatment groups. We summarize our findings as follows.

Firstly, on the basis of a fixed matching ratio, the test of a good propensity score model is the degree to which it results in the baseline covariates being balanced between treatment and control subjects [3]. However, it should not be expected that perfect balance will be achieved for all baseline variables between treatment and control subjects in the matched sample. Currently there is no balance test widely recognized for PSM of more than two treatments. In this study, the standardized difference was used for pairwise comparison among three groups, and the greatest standardized difference was chosen to evaluate the overall balance. If the greatest standardized difference is less than 0.1, it represents a meaningful balance among the three groups. Balance of covariates between groups was achieved when the pre_matching ratio of subjects was 1∶2∶3 or 2∶3∶5. When the pre_matching ratio of subjects was 1∶2∶7, the standardized differences were not reduced to below 0.1(but were below 0.15). The reason may be that the sample size proportion of group-1 was too small. A standardized difference below 0.1 suggests negligible bias in a given covariate between two treatments. Whether this criterion is suitable for multiple treatments is a valuable question worth further discussion.

Secondly, in spite of the pre_matching ratio of subjects, the matching ratio increased as caliper width increased. When the caliper width changed from 0.1 to 0.2 of the pooled standard deviation of the logit of the propensity score, the increase in matching ratio was substantial (increased by 12.4%, 8.8%, and 9.7% when the pre_matching ratios of subjects were 1∶2∶7, 1∶2∶3, or 2∶3∶5, respectively). When we used caliper widths from 0.2 to 0.8 of the pooled standard deviation of the logit of the propensity score in increments of 0.1, the matching ratios were only slightly lower than that of the highest matching ratio.

Thirdly, when the pre_matching ratio of subjects was 1∶2∶7, the MSE was only slightly lower than that of the highest MSE when we used caliper widths of 0.2 of the pooled standard deviation of the logit of the propensity score. When the pre_matching ratio of subjects was 1∶2∶3 or 2∶3∶5, using a caliper width 0.2 of the pooled standard deviation of the logit of the propensity score resulted in the lowest MSE or close to the lowest MSE. This shows that using this caliper results in greater precision than achieved with other calipers. Regardless of the pre_matching ratio of subjects, using a caliper width of 0.1 of the pooled standard deviation of the logit of the propensity score resulted in the lowest bias, followed by the caliper width of 0.2 of the pooled standard deviation of the logit of the propensity. Depending on the pre_matching ratios of subjects, our findings suggest that a width of 0.2 of the standard deviation of the logit of the propensity score is the optimal caliper.

There are certain limitations to the current study. Whether the greatest standardized difference among “pairwise comparison between three groups” was a suitable indicator for overall balance is worthy of further discussion. In our simulations, only continuous outcome variables were generated in the model to determine the optimal caliper width by estimating their differences in means, but not differences in risk (for binary outcomes). In our scenarios, the first four covariates (*C*_1_–*C*_4_) were assumed to be independent normal random variables, while the last four covariates (*D*_1_–*D*_4_) were assumed to be independent Bernoulli random variables, we didn't consider the difference between continuous variables and dichotomous variables as covariates. Additional pre_matching ratios of subjects and further iterations were not examined, due to computational limitations. In our scenarios, we used approximately 10 days on a PC (CPU: Pentium Dual-Core E5400; memory: 4G). Much of the time was spent in forming the multiple propensity score matched samples.

Our studies have provided practical solutions for the application of PSM to three treatment groups or more, however, it seems not a good choice to apply PSM with treatment arms more than 4, due to the insufficiently overlapped regions among groups, computational complexity and burden.
